# Iron Homeostasis at High Altitude in Patients With Pulmonary Hypertension

**DOI:** 10.1002/pul2.70352

**Published:** 2026-07-14

**Authors:** Aurelia E. Reiser, Markus Thiersch, Max Gassmann, Simon R. Schneider, Laura Mayer, Stéphanie Saxer, Michael Furian, Richard Sparla, Esther I. Schwarz, Mona Lichtblau, Martina U. Muckenthaler, Silvia Ulrich

**Affiliations:** ^1^ Institute of Veterinary Physiology Vetsuisse Faculty, University of Zürich Zurich Switzerland; ^2^ Clinic of Pulmonology University Hospital of Zurich Zurich Switzerland; ^3^ Eastern Switzerland University of Applied Sciences St. Gallen Switzerland; ^4^ Department of Pediatric Hematology, Oncology, and Immunology, University of Heidelberg, Heidelberg, Germany, Molecular Medicine Partnership Unit (MMPU), European Molecular Biology Laboratory and University of Heidelberg, Heidelberg, Germany, German Centre for Cardiovascular Research (DZHK), Partner site Heidelberg/Mannheim, Heidelberg, Germany, Translational Lung Research Center Heidelberg (TLRC), German Center for Lung Research (DZL) University of Heidelberg Heidelberg Germany

**Keywords:** chronic thromboembolic pulmonary hypertension, hypoxia, pulmonary arterial hypertension, transferrin

## Abstract

Iron deficiency aggravates hypoxic pulmonary vasoconstriction, exacerbating the increase of pulmonary arterial pressure at high altitude (HA). This may be especially relevant for patients with pulmonary hypertension (PH) travelling to HA, who moreover have a high prevalence of iron deficiency. Currently, no data is available on iron parameters and their influence on HA adaptation in PH‐patients. In a randomized cross‐over trial, 27 patients (44% female, mean age 61.7 ± 13.6 y) with pulmonary arterial hypertension (67%) or chronic thromboembolic PH (33%) were assessed at baseline (Zurich, 470 m) and during a stay at 2500 m. Blood samples were taken at baseline and after 20 h at 2500 m. A significant increase in ferritin (131 ± 68 to 140 ± 75, *p* = 0.002), transferrin (28.0 ± 4.7 to 30.3 ± 4.4, *p* = 0.012) and soluble transferrin receptor (sTfR) levels (2.7 ± 0.7 to 2.9 ± 0.6, *p* = 0.014) was observed at HA, all of which correlated with decreasing hepcidin levels. Arterial partial pressure of carbon dioxide (PaCO_2_) was inversely correlated with transferrin levels (Pearson's *r *= −0.57, *p* < 0.001), and baseline transferrin concentration was an independent predictor of PaCO_2_ at baseline and HA (*p* = 0.037). Furthermore, transferrin levels < 30 µmol/L were an independent predictor of mean nocturnal oxygen saturation (SpO_2_) at baseline and HA. The need for oxygen supplementation at HA could be predicted using a model including baseline transferrin, achieving a positive predictive value of 84%, suggesting that transferrin may serve as a useful clinical marker to identify PH patients at risk of oxygen desaturation at HA.

## 1 Introduction

More than 100 million people travel to areas above 2500 m every year, exposing themselves to hypobaric hypoxia [1]. This is associated with risks such as severe hypoxemia, acute mountain sickness, or, with increasing altitude, also high altitude cerebral or pulmonary oedema [2]. Whilst in healthy people adverse events usually occur at altitudes far above 2500–3000 m, patients with cardiopulmonary diseases may be affected even at lower altitudes [2]. Hence, they may also experience adverse events when travelling by airplane, even though the maximum cabin pressure is limited to altitude equivalents of 2438 m by authorities [3].

Hypoxia leads to pulmonary vasoconstriction and thus, along with an increased heart rate and temporarily increased cardiac output, to an increase in pulmonary arterial pressure [4, 5, 6, 7]. With these changes seen also in healthy people, this may be critical for patients with pulmonary hypertension (PH), in which pulmonary arterial pressure is already elevated. Clinical trials have shown that in patients with stable PH mean oxygen saturation (SpO_2_) decreases on average by 5%–7% at 2500 m, with severe hypoxemia (defined as < 80% for > 30 min) being experienced by 11% of patients during a day‐long stay and 37% of patients during an overnight stay at 2500 m [8, 9]. Echocardiographically, a significant increase in systolic pulmonary arterial pressure and a decrease in right ventricular arterial coupling was seen [9, 10].

For clinicians counselling PH patients on air travel or high‐altitude stays, having a tool to identify patients at risk of adverse events or in need of oxygen supplementation would be beneficial. However, data on potential predictors are scarce. A study by Schneider et al. identified baseline New York Heart Association (NYHA) functional class as a potential predictor of severe hypoxemia requiring oxygen supplementation at 2500 m, with functional class III having a sensitivity of 67% and specificity of 89% [11]. In patients with chronic obstructive pulmonary disease, hypoxia altitude simulation testing at low altitude has been shown to predict severe hypoxemia at high altitude, but not necessarily adverse events [12].

Hypoxia at high altitude has been associated with changes in blood parameters of iron homeostasis [13]. Over 48 h, levels of the intracellular iron‐binding protein ferritin decrease, while levels of the serum iron‐binding protein transferrin increase [14, 15]. Ferritin levels correlate with hepcidin levels, a hormone that promotes degradation of the cellular iron exporter ferroportin, potentially reflecting elevated iron release from storage organs under hypoxic conditions [13, 16]. The regulation of hepcidin during hypoxia is not fully understood, with multiple factors being involved. One such factor is erythropoietin (EPO), which stimulates erythroferrone (ERFE) secretion, leading to suppression of hepcidin [13].

Interestingly, iron deficiency leads to a stronger increase of pulmonary artery systolic pressure in hypoxia and intravenous iron administration can attenuate the pressure rise by around 50% even in non‐deficient subjects [4, 5, 6]. Iron deficiency is common in PH patients and is associated with a worse prognosis and higher mean pulmonary arterial pressure [17, 18, 19]. As PH patients already present with higher baseline pulmonary arterial pressure and are prone to iron deficiency, the question arises whether iron homeostasis influences adaptation to high altitude in these patients. However, no data is currently available on the role of iron parameters on altitude tolerance in PH patients.

Hence, in this study we assessed how iron parameters (including hepcidin, EPO and ERFE) change between baseline and an overnight high‐altitude exposure in patients with stable pulmonary arterial hypertension (PAH) or distal chronic thromboembolic PH (CTEPH) living at low altitude, and whether they have an influence on altitude tolerance, as measured by parameters of oxygenation and exercise testing. Additionally, we evaluated whether iron parameters could serve as predictors of the need for oxygen supplementation at altitude in PH patients.

## 2 Methods

### 2.1 Study Design

In this randomized cross‐over trial performed between October 2021 and February 2022 patients with PAH or distal CTEPH (inoperable or persistent after pulmonary endarterectomy) were assessed at 470 m (University Hospital Zurich, Zurich, Switzerland) and in the morning after having stayed over 20 h overnight at 2500 m (Mount Säntis, Switzerland). Patients were either first assessed at baseline (470 m) or at high altitude (2500 m), depending on randomization sequence, with a minimum washout period of 2 weeks between measurements. Randomization was performed by a software in blocks of random sizes. Patients with unstable PH, concomitant severe diseases, pregnancy, other forms of PH or with severe hypoxemia (partial arterial oxygen pressure < 7.3 kPa) at low altitude were excluded. Patients who had stayed for more than three nights at an altitude > 1000 m within the last 4 weeks before study participation were not included.

This study is part of a larger study and several results have been published previously [8, 10, 20, 21]. In this study, we focused on iron parameters in PH patients at altitude which have not been reported before.

### 2.2 Ethics

All patients provided written informed consent and the study was approved by the local ethics committee (Registration number 2021‐00243). The study was registered on clinicaltrials.gov (NCT05107700).

### 2.3 Assessments/Interventions

Patients travelled by cable car to 2500 m and stayed at this altitude overnight for around 30 h. For safety reasons, SpO_2_ and overall condition of patients were closely monitored and supplemental oxygen was administered in case of an SpO_2_ < 80% for > 30 min. At low and high altitude, various assessments were performed, including cardiopulmonary exercise testing (CPET), overnight sleep study with continuous SpO_2_ monitoring, and blood sampling. CPET was performed in the afternoon of the first day at altitude and at baseline following the ATS guidelines [10, 20, 22]. Respiratory sleep studies were performed from approximately 10 p.m. to 6 a.m., recording nasal pressure, respiratory effort, pulse oximetry, body position, and electrocardiogram [21].

Serum samples were drawn at baseline and in the morning of the second day at altitude after approximately 20 h at 2500 m, and subsequently stored at −80°C. Iron parameters including iron, ferritin, transferrin, and soluble transferrin receptor (sTfR), as well as EPO were analysed at the Institute of Clinical Chemistry, University Hospital of Zurich. ERFE (SKU# ERF‐001, Intrinsic Lifesciences, La Jolla CA, USA) and hepcidin (EIA‐5782, DRG Diagnostics, Springfield NJ, USA) were measured using ELISA assays according to manufacturer's protocol at the University of Heidelberg.

### 2.4 Statistics

Sample size was determined based on the main trial [8]. Changes of parameters between baseline and altitude were assessed using mixed models with intervention‐period interaction and correcting for age, sex, and PH group. The fulfilment of the assumptions for linear models was checked graphically. ERFE, EPO, CRP, and sTfR values were log transformed and the square root transformation was used for ferritin in the mixed regression models. When constructing the predictive model, forward selection by Akaike Information Criteria was employed to select parameters included in the final model. Assumptions of the mixed model were assessed graphically, and overdispersion, singular model fits, and collinearity were also examined. Statistical analysis was conducted using R Studio, Version 2024.04.2. Statistical significance was considered when the *p*‐value was < 0.05.

## 3 Results

Of 65 patients assessed for eligibility, 27 patients, 44% female, with a mean age of 61.7 ± 13.6 years were included in the study (Table 1) [20]. Approximately three quarters of patients had PAH, the rest had distal CTEPH. 10 patients (37%) had iron deficiency according to the current definition of the European Society of Cardiology, none of the patients was anaemic [23, 24].

**Table 1:** Baseline characteristics.

	Patients (*n* = 27)
Age (years)	61.7 ± 13.6
Sex (females)	12 (44%)
PH classification	
Pulmonary arterial hypertension	20 (74%)
Distal chronic thromboembolic PH	7 (26%)
NYHA classification	
NYHA I	7 (26%)
NYHA II	19 (70%)
NYHA III	1 (4%)
6 min walking distance (m)	590 ± 80
Mean pulmonary arterial pressure (mmHg)	42 ± 14
Pulmonary vascular resistance (WU)	5.9 ± 2.7
Iron deficiency*	10 (37%)
Anemia	0 (0%)

*Note:* Results are presented as mean ± standard deviation or as number (%). *Iron deficiency was defined as ferritin < 100 μg/L or ferritin 100–299 μg/L + TSAT < 20% (current ESC definition) [23]. Anemia was defined as hemoglobin < 12 g/dL for females and < 13 g/dL for males.

Abbreviations: NYHA, New York Heart Association classification; PH, pulmonary hypertension.

After 20 h at 2500 m, ERFE and EPO levels significantly increased (*p* < 0.001) and hepcidin was downregulated (*p* = 0.001) after correction for age, sex, and PH‐diagnostic group (Table 2). Ferritin levels (*p* = 0.002), as well as transferrin and sTfR levels (*p* < 0.01) increased at altitude, whilst the transferrin receptor index (sTfR/log(ferritin)) did not change (Table 2).

**Table 2:** Change of iron parameters after 20 h at altitude compared to baseline.

	Baseline (470 m)	Altitude (2500 m)	*p* value
Iron parameters			
Iron (µmol/L)	20.0 ± 4.9	18.0 ± 6.7	0.333
Ferritin (µg/L)	131 ± 68	140 ± 75	**0.002**
Transferrin (µmol/L)	28.0 ± 4.7	30.3 ± 4.4	**0.012**
sTfR (mg/L)	2.7 ± 0.7	2.9 ± 0.6	**0.014**
TSAT (%)	36 ± 10	30 ± 11	0.057
Transferrin receptor index	0.6 ± 0.3	0.6 ± 0.2	0.767
CRP (mg/L)	1.5 ± 0.9	1.6 ± 1.3	0.817
Hepcidin (ng/mL)	15.0 ± 5.5	11.1 ± 3.2	**0.001**
ERFE (ng/mL)	1.4 ± 1.7	2.2 ± 1.9	**< 0.001**
EPO (IU/l)	13.5 ± 9.2	31.8 ± 22.7	**< 0.001**
Arterial blood gas analysis at rest/nocturnal SpO_2_			
Haemoglobin (g/dL)	14.7 ± 1.5	14.6 ± 1.1	0.649
Haematocrit (%)	45 ± 4	45 ± 3	0.702
PaO_2_ (kPa)	10.0 ± 1.7	7.9 ± 1.4	**< 0.001**
PaCO_2_ (kPa)	4.6 ± 0.5	4.5 ± 0.5	**0.001**
SaO_2_ (%)	95.1 ± 2.9	91.2 ± 4.2	**0.001**
pH	7.43 ± 0.04	7.46 ± 0.02	**0.011**
Bicarbonate (mmol/L)	24.1 ± 2.3	25.0 ± 1.6	0.644
Mean nocturnal SpO_2_ (%)	91 ± 2	83 ± 4	**< 0.001**
Cardiopulmonary exercise testing outcomes (end‐exercise)			
Minute ventilation (l/min)	72.1 ± 3.7	74.5 ± 3.1	0.686
Tidal volume (l)	2.0 ± 3.7	2.0 ± 3.1	0.194
VE/VCO_2_	40 ± 4	44 ± 3	**0.001**
PETCO_2_ (mmHg)	22 ± 4	20 ± 3	**< 0.001**

*Note:* Results are presented as mean ± standard deviation. Bold values indicate statistical significant at (*p* < 0.05). Statistical analysis was performed using a linear mixed model accounting for age, sex, PH aetiology and period‐intervention‐interaction.

Abbreviations: CRP, C‐reactive protein; EPO, erythropoietin; ERFE, erythroferrone; PaCO_2_, arterial partial carbon dioxide pressure; PaO_2_, arterial partial oxygen pressure; SaO_2_, arterial oxygen saturation; sTfR, soluble transferrin receptor; TSAT, transferrin saturation; VE/VCO_2_, respiratory equivalent for carbon dioxide; PETCO_2_, end‐tidal partial pressure of carbon dioxide.

Of note, ERFE values from 2 patients were over 10‐fold higher than the rest of the patients, both at baseline and altitude. One patient had elevated CRP levels most probably explaining the markedly higher ferritin levels. The above‐mentioned samples were excluded from further analysis of the respective parameters (ERFE/Ferritin) as they led to a violation of the assumptions of linear mixed models.

**Figure 1:**
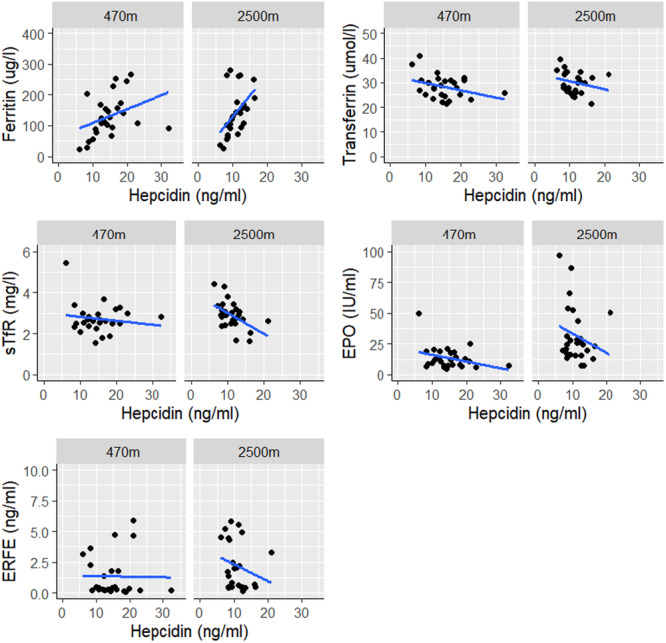
Correlation between iron parameters and hepcidin levels at baseline (470 m) and high altitude (2500 m). EPO, erythropoietin; ERFE, erythroferrone; sTfR, soluble transferrin receptor.

As seen in Figure 1, ferritin levels positively correlated with hepcidin (Pearson's *r* of 0.31, *p* = 0.026), and transferrin (Pearson's *r* of −0.35, *p* = 0.009) as well as sTfR (Pearson's *r* −0.30, *p* = 0.03) negatively correlated with hepcidin. Furthermore, there was a negative correlation between hepcidin and EPO levels (Pearson's *r* of −0.36, *p* = 0.008), especially at high altitude. However, ERFE levels did not correlate with hepcidin (Pearson's *r* of −0.18, *p* = 0.209). Extended analysis using a mixed linear model correcting for location, age, sex, and PH diagnostic group showed that an increase in EPO by 10 IU/L led to an average decrease in hepcidin by 1.3% (*p* = 0.049), with the decrease tending to be stronger at altitude (*p* = 0.07).

### 3.1 Correlation of iron Parameters With Arterial Blood Gas Parameters

At high altitude, arterial partial pressure of oxygen (PaO_2_) decreased significantly from 10.0 ± 1.7 kPa to 7.9 ± 1.4 kPa (*p* < 0.001), with arterial oxygen saturation dropping from 95% ± 2% to 91 ± 2% (*p* = 0.001). Arterial partial pressure of carbon dioxide (PaCO_2_) decreased from 4.6 ± 0.5 kPa to 4.5 ± 0.5 kPa (*p *= 0.001) and pH increased from 7.43 ± 0.04 to 7.46 ± 0.02 (*p* = 0.011), with no significant change of bicarbonate (*p* = 0.644), hence indicating acute respiratory alkalosis (Table 2).

**Figure 2:**
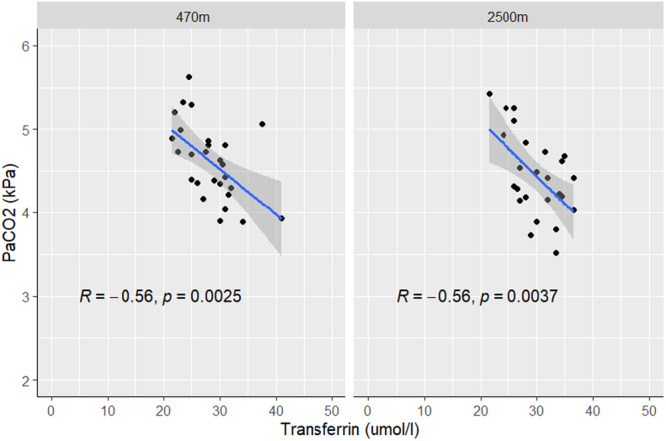
Correlation of transferrin levels to arterial partial pressure of carbon dioxide. PaCO_2_, arterial pressure of carbon dioxide.

PaCO_2_ showed a negative correlation with transferrin levels, both at baseline and at altitude (Figure 2) with an overall Pearson's *r* of −0.56 (*p* < 0.001). Transferrin concentration was an independent predictor of PaCO_2_ at baseline and high altitude after correction for sex, age and CRP levels (*p* = 0.037). None of the other iron parameters (iron, ferritin, sTfR, EPO, ERFE, hepcidin) showed a clear correlation to either PaO_2_ or PaCO_2_, so no further statistical analyses were performed (data not shown).

### 3.2 Correlation of Iron Parameters With Parameters of Cardio‐Pulmonary Exercise Testing

**Figure 3:**
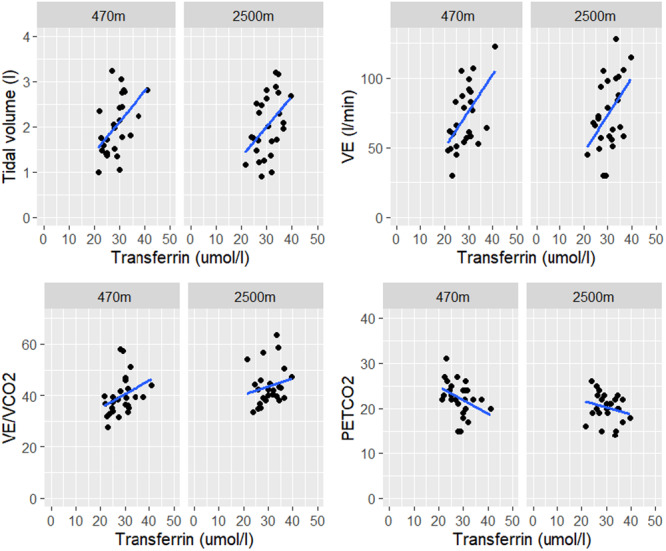
Correlation of transferrin levels to parameters of cardiopulmonary exercise testing at baseline and altitude. PETCO2, end‐tidal partial pressure of carbon dioxide; VE, minute ventilation; VE/VCO2, respiratory equivalent for carbon dioxide.

At high altitude, end‐exercise respiratory equivalent for carbon dioxide (VE/VCO_2_) increased from 40 ± 4 to 44 ± 3 (*p* = 0.001) and end‐tidal partial pressure of carbon dioxide (PETCO_2_) decreased from 22 ± 4 to 20 ± 3 (*p* < 0.001). End‐exercise minute ventilation and tidal volume remained unchanged (*p* > 0.05). Transferrin levels moderately correlated with VE/VCO_2_ both at baseline and at altitude (Figure 3) with an overall Pearson's *r* of 0.30 (*p* = 0.03) and negatively correlated with PETCO_2_ (Pearson's *r* of −0.35, *p* = 0.01). Further analysis showed that higher transferrin levels correlated with a higher minute ventilation (Pearson's *r* of 0.50, *p* < 0.001) and a higher tidal volume (Pearson's *r* of 0.46, *p* = 0.001); however, transferrin was not an independent predictor of these parameters after correction for sex, age and PH category.

### 3.3 Baseline Transferrin, Nocturnal Oxygen Saturation and Oxygen Supplementation

The main predefined adverse event occurring at high altitude was severe nocturnal oxygen desaturation (SpO_2_ < 80% for > 30 min.), which mainly occurred during the night [8].

Mean nocturnal SpO_2_ strongly correlated with the need for oxygen supplementation, not only at night (Chi‐squared *p* = 0.002), but also during the whole stay at altitude (*p* < 0.001), with a mean nocturnal SpO_2_ < 82% having a positive predictive value of 89.5% for oxygen supplementation at high altitude.

As transferrin correlated with resting PaCO_2_ and end‐exercise CPET ventilation parameters at baseline and altitude, the suitability of transferrin to predict nocturnal SpO_2_ and hence the need to supplement oxygen at altitude was assessed. All patients with a nocturnal SpO_2_ < 80% for > 30 min had baseline transferrin levels less than 30 µmol/L. Transferrin levels below 30 µmol/L at baseline were an independent predictor of mean nocturnal SpO_2_ at baseline and high altitude in a linear mixed model correcting for age, sex and PH aetiology with mean nocturnal SpO_2_ decreasing by 2.8 percentage points with a low transferrin independent of location (*p* = 0.011).

**Figure 4:**
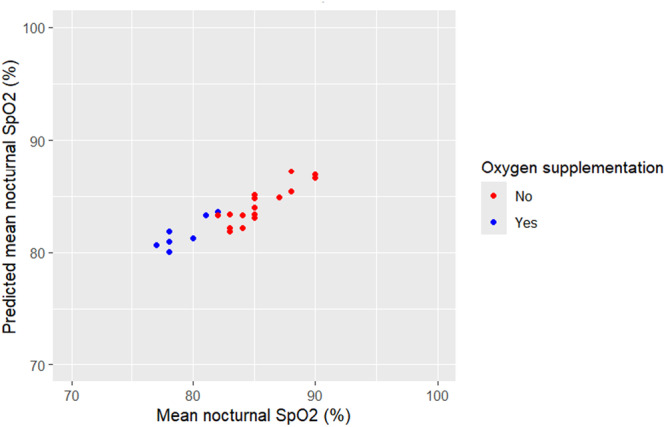
Predicted nocturnal oxygen saturation versus measured SpO_2_. SpO_2_, peripheral oxygen saturation.

In a next step, a predictive mixed linear model was constructed by forward selection using location and low baseline transferrin as parameters to predict nocturnal oxygen saturation. As age, sex and PH aetiology did not improve the model, they were not included. This model had a marginal *R* ^2^ of 0.68 and a conditional *R* ^2^ of 0.81. As depicted in Figure 4, predicted and measured nocturnal SpO_2_ at altitude were closely related, with a mean absolute error of 1.38 and a mean squared error of 2.98. Using the mean nocturnal SpO_2_ cut‐off determined in the original data of 82%, oxygen supplementation could be predicted indirectly by baseline transferrin with a positive predictive value of 84%, a sensitivity of 94%, a negative predictive value of 86% and a specificity of 67%.

## 4 Discussion

In this study, we could show that in patients with stable PAH or distal CTEPH changes in iron parameters upon acute overnight exposure to high altitude reflect a strong erythropoietic effect of hypoxia and a tendency to iron deficiency. Transferrin levels were an independent predictor of PaCO_2_ as a marker of excessive ventilation. Transferrin < 30 µmol/L was an independent predictor of mean nocturnal oxygen SpO_2_, indirectly predicting the need for oxygen supplementation due to severe hypoxemia at high altitude.

In healthy people travelling to high altitude, previous studies have reported a short‐term (< 24 h) increase of ferritin and an increase of transferrin, with ferritin correlating with hepcidin levels [14, 15, 16]. Of note, those participants were younger (median age 33–44 years) and altitudes were higher, but baseline iron parameters were comparable, none of the study participants were iron deficient, and in 2 of 3 studies the sex distribution was comparable to our study. We observed the same changes in PH patients. As no significant increase in hematocrit was observed in our study, we deemed the influence of decreasing plasma volume at high altitude (as described previously in healthy people) on the observed changes in iron parameters low [25]. The slight elevation of ferritin could potentially be explained by mild inflammation, even though we did not see a significant change in CRP. However, interleukin‐6 can increase at higher altitude in healthy with no changes in CRP levels, albeit this was not measured in our study [26]. As inflammation can hinder the use of iron for erythropoiesis despite adequate iron stores; hence, leading to functional iron deficiency, this could also be a possible explanation why intravenous iron substitution in non‐iron deficient healthy people had no effect on EPO levels at altitude and hepcidin levels eventually reached the same levels as those of participants without iron substitution [4, 14]. Additionally to the increase in transferrin levels, we saw a significant increase in sTfR already after 20 h at 2500 m, even though a previous study in healthy people did not report a significant change in sTfR until 144 h at 4340 m [14]. Elevated transferrin and sTfR levels are an indicator of iron deficiency, both for absolute deficiency and functional deficiency, that is, iron deficiency in tissues despite adequate iron storage [27]. The relatively early increase in sTfR in PH patients compared to healthy individuals may hence reflect either a stronger erythropoietic response to hypoxia and/or a faster development of iron deficiency in PH patients compared with healthy individuals. This could also explain the correlation between changes in hepcidin levels and changes in EPO levels in our study, in contrast to a previous study in healthy subjects reporting no correlation [14]. From a mechanistical view, both iron deficiency and hypoxia inhibit prolyl hydroxylase 2, hence decreasing degradation of the hypoxia‐inducible factor‐2 alpha subunit (HIF‐2α), which after heterodimerization enhances EPO transcription [13]. However, HIF2α also has a 5′‐iron responsive element (IRE), allowing negative feedback control by binding of iron regulatory protein (IRP) in case of low iron [28]. Of note, hypoxia also influences IRP binding activity, with IRP1 binding being decreased (unless treated with iron chelator), but IRP2 increased [13, 29, 30]. Furthermore, Tfr1 has a hypoxia responsive element in its promoter region [31]. Besides these pathways, there may also be other pathways involved such as the Kelch‐like ECH‐associated protein 1/NF‐E2 p45‐related factor 2 (KEAP1/NRF2) pathway [32]. From a functional viewpoint, all measures seem to increase cellular iron uptake to meet increased demands during hypoxia. If this demand cannot be met, or iron is not released from the storage, this might lead to absolute/functional iron deficiency, which is not uncommon in PH patients. Upregulation of transferrin and Tfr1 in the blood is a good indicator of absolute or functional iron deficiency, and it has been shown that iron deficiency can exaggerate the hypoxic response. Hence, it may be very difficult to distinguish between a purely enhanced hypoxic response, a purely stronger tendency to iron deficiency, or a combination of the above. More data from preclinical trials and studies designed to investigate this question are necessary to confirm this hypothesis.

In this study, transferrin was an independent predictor of PaCO_2_, with higher transferrin levels correlating to lower PaCO_2_. Furthermore, transferrin positively correlated with minute ventilation and tidal volume during cardiopulmonary exercise testing, although transferrin was not an independent predictor of these parameters. These findings could suggest that higher transferrin levels correlate with increased ventilation at altitude, that is, higher transferrin levels lead to stronger hypoxia‐induced hyperventilation. However, there is no data from healthy people to confirm these findings and there is no proven biological explanation. A study performed in patients with anaemic, iron‐deficient chronic heart failure reported a reduction of central chemoreflex sensitivity after intravenous iron substitution, but comparisons are hampered because of the differences between the study populations regarding anemia, iron deficiency, and underlying disease [33]. Furthermore, as only transferrin but none of the other iron parameters correlated with ventilation parameters, the link between ventilation and transferrin may not lie in iron availability, but in a common denominator of both. A possible factor may be hypoxia‐inducible factor 1α (HIF‐1α), which is upregulated under hypoxia [34]. Partial deficiency of HIF‐1α and thus of the HIF‐1 heterodimer leads to impaired ventilatory response during chronic hypoxia in mice [35]. HIF‐1 can upregulate transferrin through direct binding to its promoter region [34]. Hence, higher transferrin levels potentially may reflect upregulated HIF‐1 levels, which lead to a stronger hypoxic ventilatory response.

This hypothesis may also explain why baseline transferrin levels below 30 µmol/L were an independent predictor of mean nocturnal SpO_2_ at baseline and altitude. This is of great clinical interest, as nocturnal desaturation is a strong risk factor for adverse effects of altitude, with many of these effects occurring during sleep, especially in patients with underlying pulmonary disease [36, 37]. However, this study was not designed to answer this question. If confirmed, these findings are of great clinical interest, predicting the eventual need of oxygen supplementation at 2500 m, especially as the model had a positive predictive value of 84%; hence, larger than the positive predictive value of 58.2% from a study of Schneider et al. using NYHA class III as a predictor [11].

## 5 Limitations

A major limitation of this study is the small sample size. In combination with the relatively low prevalence of iron deficiency, this limited the ability to draw firm conclusions on the influence of iron deficiency on altitude tolerance in PH patients. However, given the demanding logistics of conducting a field study in a rare disease, the sample size is considerable. The small sample size may also be the reason for the rather low correlation coefficients. The results cannot be extrapolated to all PH patients, as only stable patients with PAH and distal CTEPH in low‐risk classes were included. As we did not include healthy controls, no direct comparisons between healthy people and PH patients can be made. However, changes in iron parameters at high altitude have been reported repeatedly in a congruent manner, allowing for general comparisons. This study was not designed to develop a predictive model for oxygen supplementation. In addition, the criterion for oxygen supplementation used in this study (SpO_2_ < 80% for at least 30 min) was a safety endpoint with patients often reporting no symptoms requiring further treatment. Nevertheless, prolonged oxygen desaturation poses a risk, especially to patients with cardiovascular disease and hence the use of this cut‐off for oxygen supplementation is ethically justified. However, to validate these findings, further studies are necessary.

## 6 Conclusion

To conclude, changes in iron homeostasis during acute high altitude exposure in PH patients are comparable to those observed in healthy people. However, the earlier rise in sTfR levels may indicate a stronger erythropoietic effect of hypoxia and/or a facilitated development of iron deficiency in PH patients. Explorative analyses suggest that transferrin may be used to predict nocturnal SpO_2_, offering means to assess the need for oxygen supplementation at altitude. If confirmed in further studies, this may provide a valuable tool to counsel PH patients travelling to altitude.

## Author Contributions

A.E.R. and S.U. were involved in conceptualization, L.M., S.S., M.F., E.I.S., S.R.S, S.U., M.L. were involved in recruiting of participants and organizing sample acquisition, L.M., S.S., M.F., E.I.S., S.R.S., A.E.R. and R.S. were involved in data acquisition, R.S., S.R.S. and A.E.R. were involved in data curation, A.E.R. performed data analysis, M.U.M., M.T., M.G., M.L. and S.U. provided significant guidance on data interpretation. S.U. was involved in funding acquisition. A.E.R. (draft), M.T., M.G., M.L., M.U.M. and S.U. (review/editing) were involved in the writing process.

## Ethics Statement

All study participants provided written informed consent and this study was approved by the local ethics committee (KEK2021‐00243). S.U. takes responsibility for the integrity of the work.

## Conflicts of Interest

S.U. reports grants from Johnson and Johnson SA, Switzerland, during the conduct of the study; and grants from the Swiss National Science Foundation and Zurich Lung, grants and personal fees from Orpha Swiss, and personal fees from Actelion SA and MSD SA, outside the submitted work. M.L. reports travel grants from Johnson and Johnson SA, Switzerland, and personal fees from MSD, Switzerland, during the conduct of the study, grants from the Swiss Lung League, all outside the submitted work. The other authors declare no conflicts of interest.

## Data Availability

The data that support the findings of this study are available on request from the corresponding author. The data are not publicly available due to privacy or ethical restrictions.
